# C-755-07. Beyond the Flames: A Structured Education Approach for Enhanced Burn Care and Certification

**DOI:** 10.1093/jbcr/irag033.043

**Published:** 2026-04-06

**Authors:** Amanda Kumar

**Affiliations:** University of California San Diego Health, Murrieta, CA

## Abstract

**Introduction:**

A specialty certification in burn nursing was introduced in 2023 by a professional certification agency to recognize advanced expertise in burn care. At a regional academic burn center, only 4.5% of nurses were certified as of July 2024. Survey results revealed limited confidence and lack of resources for exam preparation, highlighting the need for structured educational support.

**Methods:**

Guided by the 8A’s Evidence-Based Practice Model, a four-hour burn certification review course was developed, incorporating lectures, powerpoint modules, practice exam questions, and test-taking strategies. Institutional support included provision of 10 exam scholarships and access to study materials. Pre- and post-intervention surveys and knowledge assessments were conducted to measure outcomes.

**Results:**

Staff confidence in passing the exam increased by 55% (n = 18). Mean pre-/post-test scores improved from 47% to 77%. Among approximately 65 eligible nurses, certification rates increased from 5% to 26%. An additional 16 nurses are currently scheduled to test, which, if successful, would increase the certification rate to nearly 50%.

**Conclusions:**

Implementation of a structured burn nursing certification review course, coupled with institutional support through scholarships and study resources, resulted in significant improvements in nurse confidence, knowledge acquisition, and certification attainment. These outcomes demonstrate the effectiveness of targeted educational interventions in advancing professional development while directly supporting burn nursing competency standards. Alignment with Magnet criteria (SE3, SE5) further underscores this initiative's contribution to nursing excellence and quality burn care delivery. Sustainability will be ensured through biannual review courses, ongoing study sessions, and mentorship, fostering a culture of continuous learning and professional achievement.

**Applicability of Research to Practice:**

This initiative demonstrates how structured educational programs can directly enhance specialty certification, elevate nursing practice standards, and improve quality of care in burn nursing. By aligning with burn nursing competency standards, the model can be adapted across burn centers and specialty units to support professional development and organizational excellence.

**Funding for the study:**

N/A.

